# Supplementary material for: Snow is an important control of plant community functional composition in oroarctic tundra
Source: Oecologia. 2019 Sep 14;191(3):601–8. doi: 10.1007/s00442-019-04508-8 (PMC6825026; doi:10.1007/s00442-019-04508-8)

Electronic supplementary material for the article "Snow is an important control of plant community functional composition in oroarctic tundra"

Konsta Happonen[1,*], Juha Aalto[1], Julia Kemppinen[1], Pekka Niittynen[1], Anna-Maria Virkkala[1], and Miska Luoto[1]

[1]Department of Geosciences and Geography, University of Helsinki

[*]Corresponding author: konsta.happonen@helsinki.fi

21-Aug-2019

Appendix S1

Table S1. List of species and their total point-intercept frequencies in the 143 surveyed plots. Nomenclature follows Tropicos (http://www.tropicos.org).

Species	n
Empetrum nigrum	1089
Vaccinium myrtillus	625
Betula nana	439
Deschampsia flexuosa	358
Phyllodoce caerulea	324
Vaccinium vitis-idaea	306
Carex bigelowii	132
Salix herbacea	75
Juniperus communis	70
Juncus trifidus	67
Nardus stricta	53
Festuca ovina	47
Vaccinium uliginosum	45
Cassiope tetragona	43
Cassiope hypnoides	36
Linnaea borealis	33
Loiseleuria procumbens	29
Lycopodium annotinum	26
Bistorta vivipara	24

Carex vaginata	18
Solidago virgaurea	18
Viola biflora	18
Antennaria alpina	14
Calamagrostis stricta	14
Carex dioica	14
Antennaria dioica	13
Anthoxanthum odoratum	12
Dryas octopetala	12
Sibbaldia procumbens	12
Carex lachenalii	9
Pedicularis lapponica	9
Carex brunnescens	8
Taraxacum sp.	8
Trientalis europaea	8
Trollius europaeus	8
Alchemilla sp.	7
Cornus suecica	6
Andromeda polifolia	5
Carex magellanica	5
Diapensia lapponica	5
Thalictrum alpinum	5
Diphasiastrum alpinum	4

Selaginella selaginoides	4
Agrostis mertensii	3
Bartsia alpina	3
Campanula rotundifolia	3
Eriophorum angustifolium	3
Gnaphalium supinum	3
Leontodon autumnalis	3
Trisetum spicatum	3
Carex bigelowii x aquatilis	2
Hieracium alpinum	2
Minuartia biflora	2
Poa alpina	2
Potentilla palustris	2
Salix glauca	2
Salix lapponum	2
Salix polaris	2
Arctostaphylos alpina	1
Astragalus alpinus	1
Calamagrostis lapponica	1
Carex rotundata	1
Equisetum sylvaticum	1
Juncus biglumis	1
Petasites frigidus	1

Potentilla crantzii	1
Ranunculus nivalis	1
Rubus chamaemorus	1
Saussurea alpina	1
Veronica alpina	1

Figure S2. A schematic map of the study area and the different environmental measurement schemes. The inset describes how the different measurement plots are positioned within one location. Aerial photographs from the National Land Survey of Finland (CC-BY).

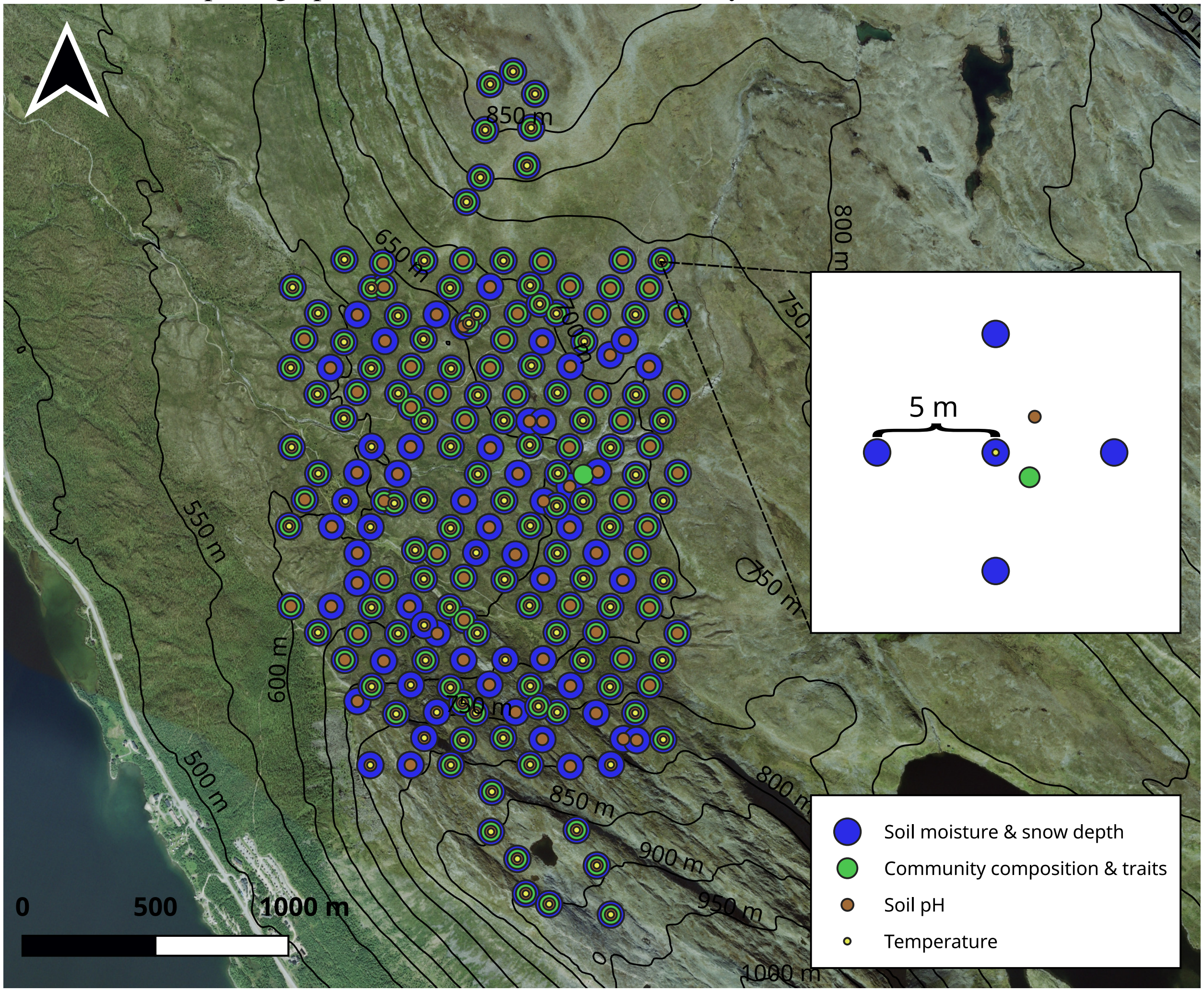

Figure S3. The point-intercept frame used in this study.

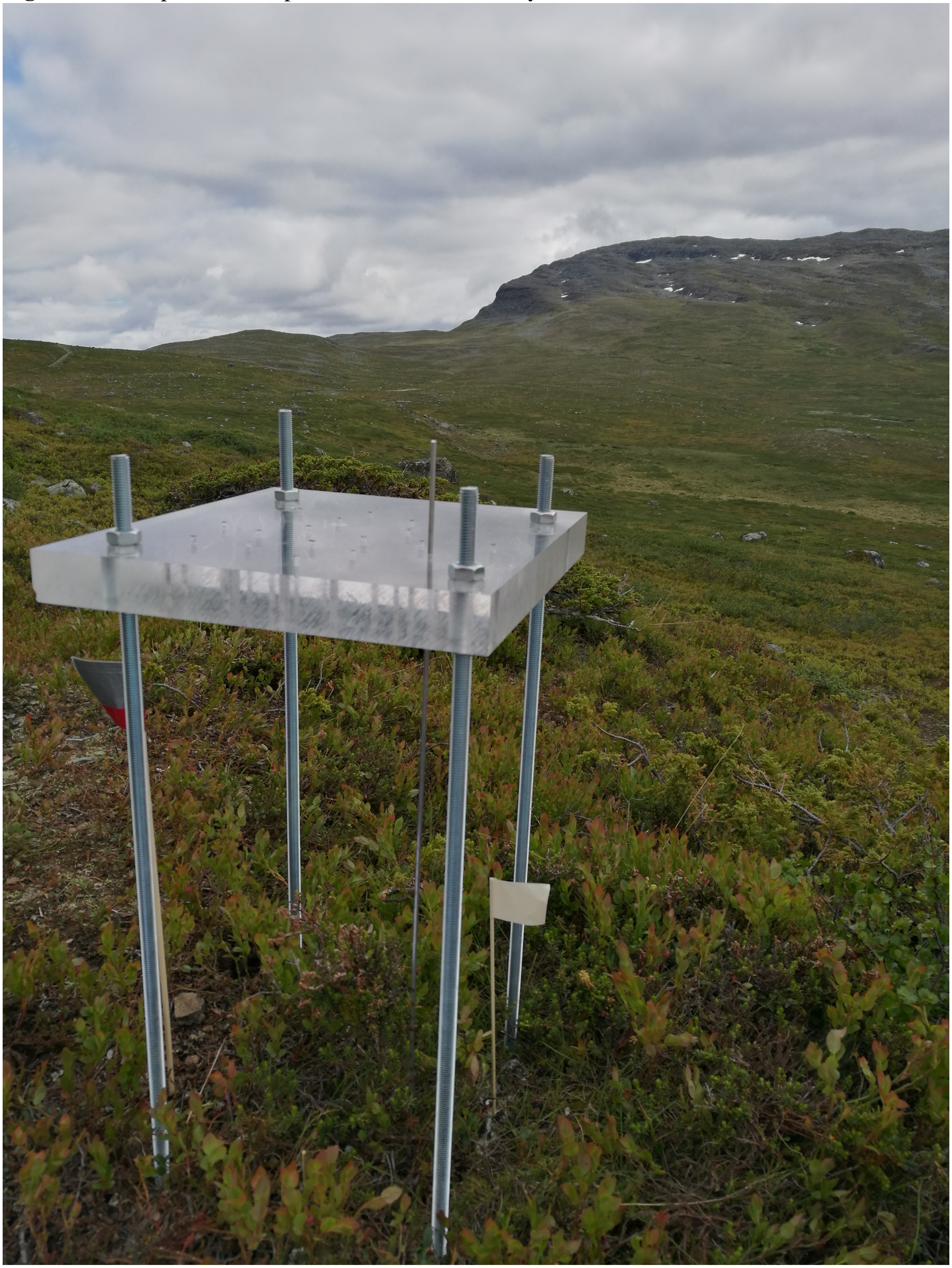

Figure S4. The relative unique contribution of July mean temperature, soil resources and maximum snow depth for explaining variance in CWM_{local}, $CWM_{landscape}$, and CWM_{global} for height, LDMC and SLA.

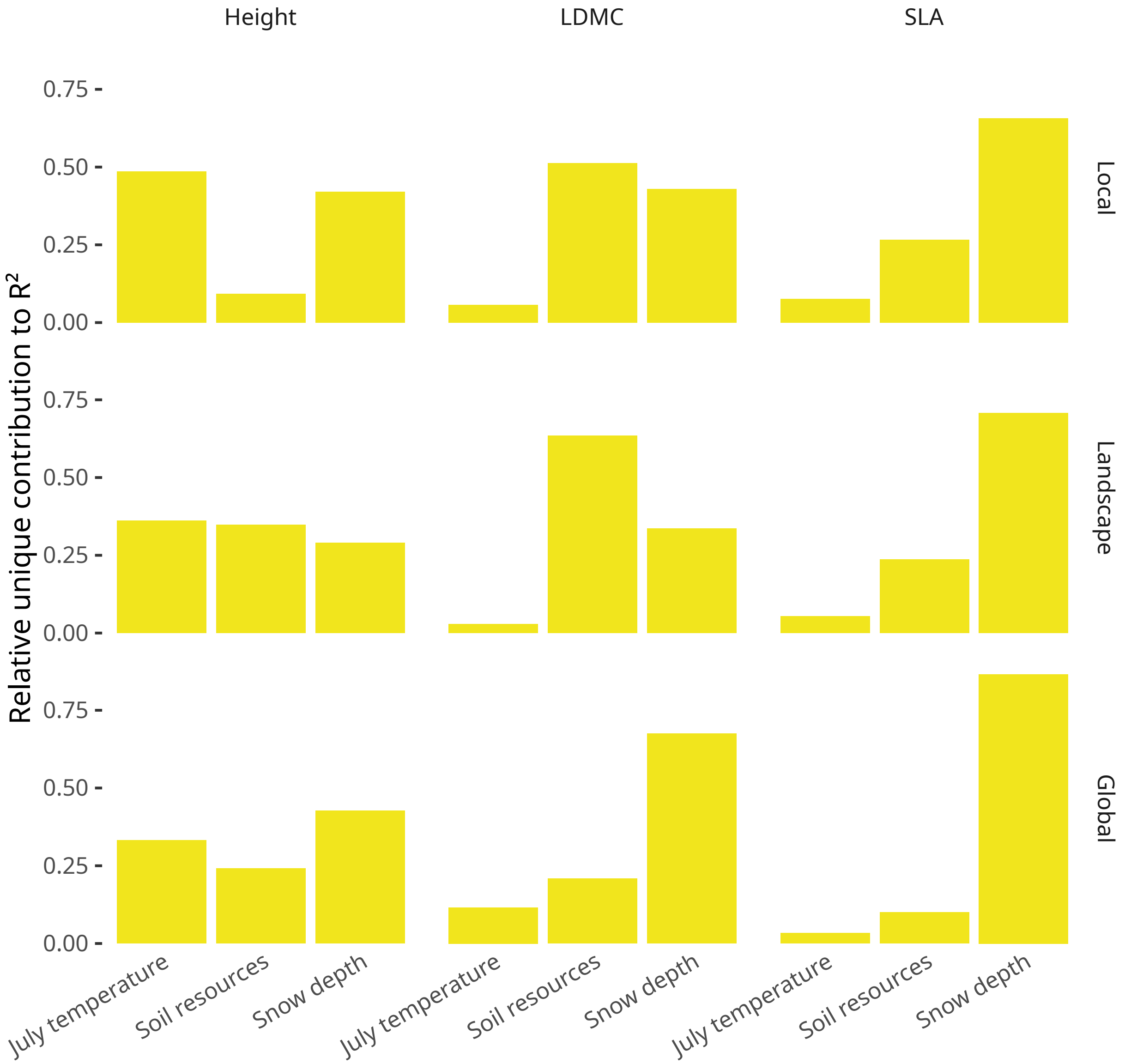

Figure S5. Smooth terms and their confidence intervals (SE*2) from models of $\log_e$-transformed CWM_{local}, $CWM_{landscape}$, and CWM_{global} for height, LDMC and SLA.

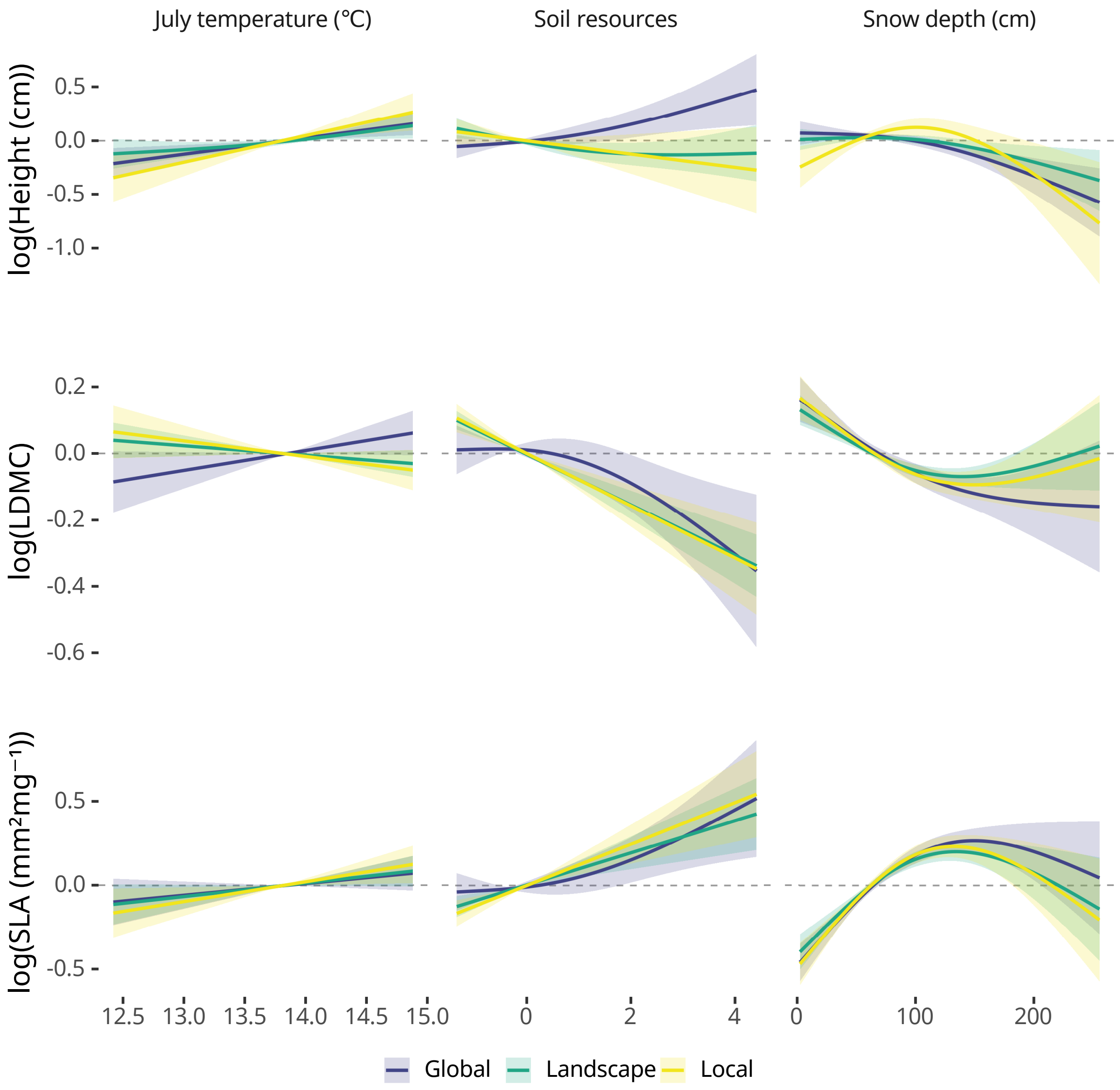

Figure S6. Covariation of $\log_e$-transformed CWM_{local}, $CWM_{landscape}$, and CWM_{global} between height, LDMC and SLA.

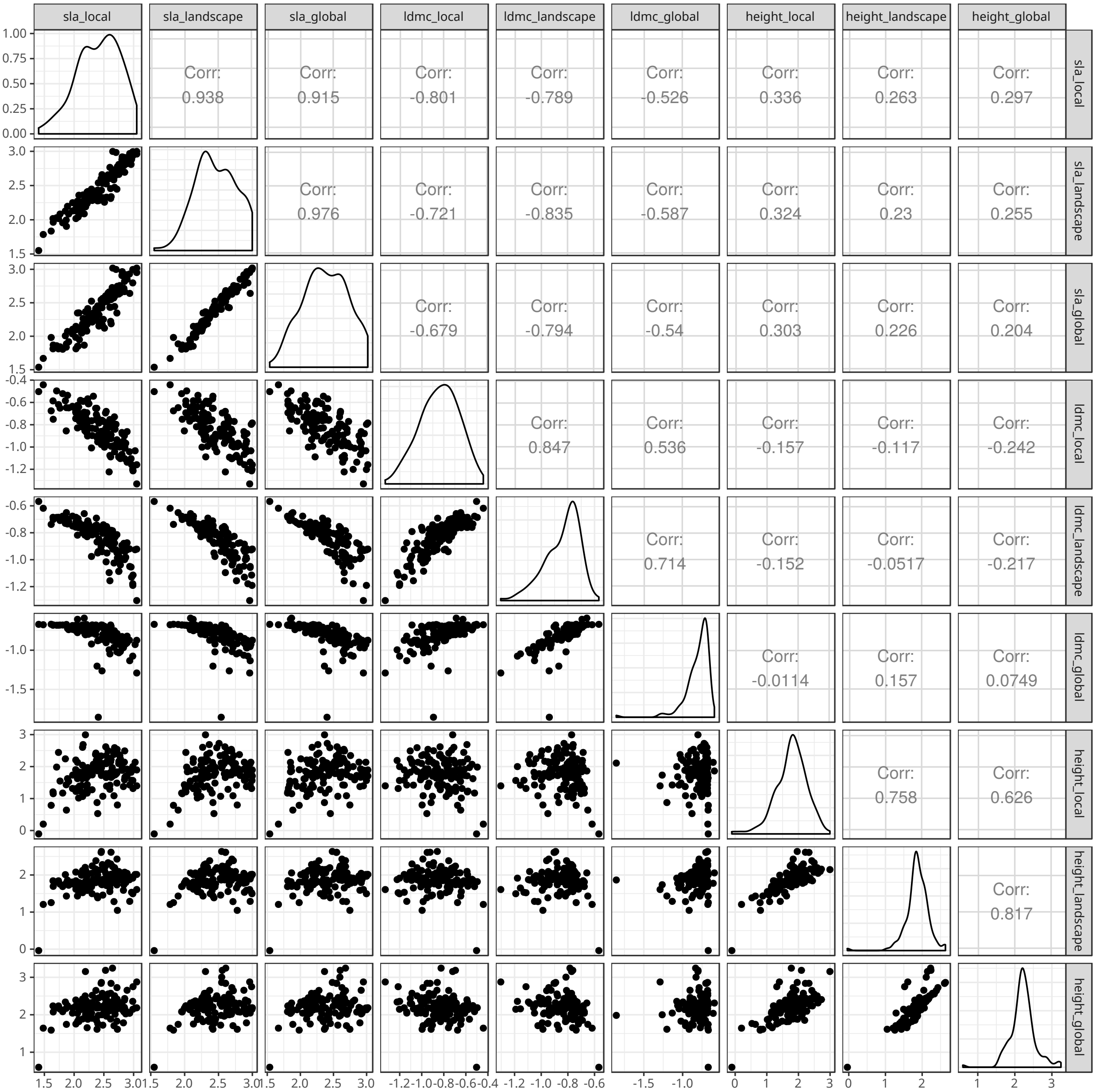

Figure S7. Covariation of environmental conditions at the study site.

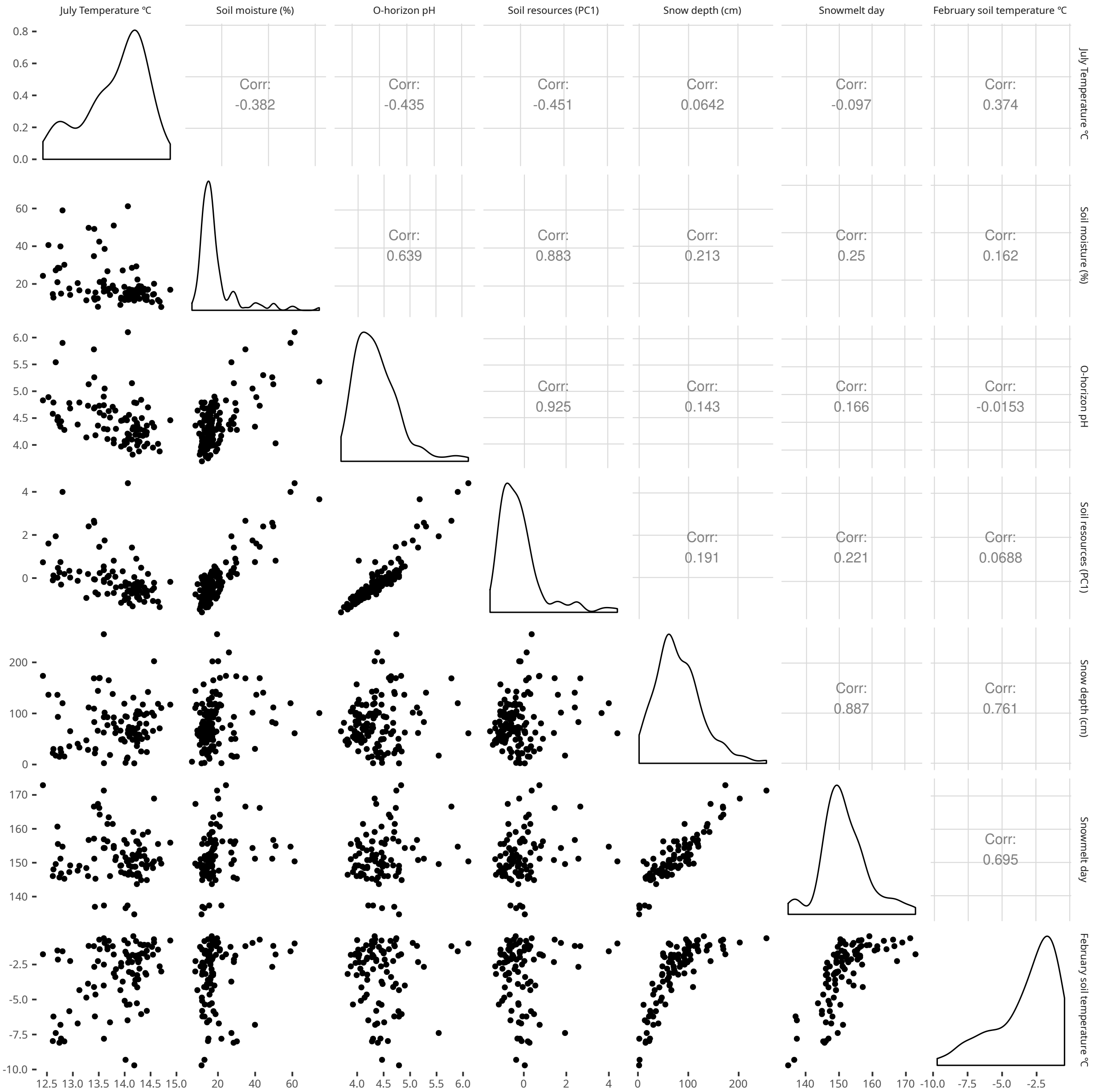

Supplement: Supplementary file 1 — Supplementary material 1 (pdf 4790 KB) [file 442_2019_4508_MOESM1_ESM.pdf]
